# Towards an HIV cure: science and debate from the International AIDS Society 2013 symposium

**DOI:** 10.1186/1742-4690-10-134

**Published:** 2013-11-13

**Authors:** Damian FJ Purcell, Julian H Elliott, Anna-Laura Ross, John Frater

**Affiliations:** 1 Department of Microbiology and Immunology, The University of Melbourne, Parkville, VIC 3010, Australia; 2 Department of Infectious Diseases, Alfred Hospital and Monash University, Prahran, VIC 3181, Australia; 3 Burnet Institute, Prahran, Australia; 4 French National Agency for Research on AIDS and viral hepatitis, ANRS, 101 rue de Tolbiac, Paris, France; 5 International AIDS Society, Geneva, Switzerland; 6 Peter Medawar Building for Pathogen Research, Oxford University, Oxford, UK; 7 Oxford NIHR Biomedical Research Centre, Oxford, UK; 8 Oxford Martin School, Oxford, UK

## Abstract

The International AIDS Society convened the multi-stakeholder “Towards an HIV Cure” symposium in Kuala Lumpur, Malaysia in 2013 to address the significant research challenges posed by the search for a cure for HIV infection. Current antiretroviral regimens select for a small reservoir of cells that harbour latent HIV provirus, produce few or no HIV virions, and resist detection or clearance by host immunity. The symposium examined basic molecular science and animal model data, and emerging and ongoing clinical trial results to prioritise strategies and determine the viral and immune responses that could lead to HIV remission without ART. Here we review the presentations that scrutinized the molecular mechanisms controlling virus expression from proviral DNA, and the intrinsic cellular restriction and immune mechanisms preventing viral production. Insights from the basic science have translated into new therapeutic strategies seeking HIV remission without ongoing therapy, and much interest was focused on these ongoing trials. We also summarise the emerging ethical issues and patient expectations as concepts move into the clinic.

## Introduction

The International AIDS Society (IAS) meetings over the past 25 years have been a focal point for optimising strategies to tackle the HIV epidemic. Antiretroviral therapy (ART) has dramatically improved HIV-associated morbidity and mortality, but is not a cure. Under the leadership of Françoise Barré-Sinoussi, co-discoverer of HIV, the IAS is focussing on achieving a safe, affordable and scalable cure for HIV infection [1].

There have been a number of high profile case reports of apparent HIV cure. The well-publicised “Berlin patient” was treated for acute myeloid leukemia with intensive conditioning to deplete his haematopoietic system ahead of restorative transplantation with homozygous CCR5∆32 bone marrow stem cells, and subsequently became aviraemic [2,3]. More recently, the cases of the “Mississippi baby” [4] and adults in the French VISCONTI cohort provide potential insights into the impact of ART when given very early in infection. Together the experiences of these individuals suggest that a cure is feasible, not withstanding the inherent challenge of eliminating every potentially replication competent provirus from the body [5].

The IAS “Towards an HIV Cure” annual symposium brings together basic scientists, clinicians, funders, and community advocates to address the key challenges facing the community in bringing about a cure for HIV. In this review, we summarise the key findings from the 2013 symposium, held in Kuala Lumpur, Malaysia on 29 and 30 June. Initially focusing on the basic science underpinning the reservoir, HIV transcription control and immune responses, the report then focuses on clinical trial and ex vivo data, before summarising the key ethical and regulatory issues.

### Molecular mechanisms restricting gene expression from HIV provirus

Investigations of non-productive HIV infection in reservoir cells have yielded important insights into molecular factors governing HIV latency [6,7]. Olivier Rohr from the University of Strasbourg presented new data on the cellular transcription factor CTIP2 from microglial cells that repress HIV through a range of interconnected activities [8]. CTIP2 binds to SP1 sites in the HIV core promoter and recruits histone deacetylases (HDACs) and the H3K9 methyltransferase, SUV39H1, resulting in repressive heterochromatin structure at the first nucleosome, Nuc1, of the viral promoter. CTIP2 synergises with LSD1, normally a lysine specific demethylase, to instead tri-methylate histone H3K4 and H3K9 at the HIV promoter, strongly repressing HIV LTR transcriptional activity. CTIP2 also associates with p-TEFb, normally a potent Tat-responsive transcription elongation factor. Intriguingly CTIP2 recruits pTEF-b into an inactive complex repressing expression from the HIV promoter. CTIP2 synergises with another repressive factor, HMGA1, in silencing HIV gene transcription. Furthermore CTIP2 serves to anchor an inactive form of p-TEFb to nascent HIV TAR RNA, and inhibits the transcription trans-activation activity of Tat. While CTIP2 may not participate in HIV repression in T-cells, it is an important HIV repressive pathway in cells of the myeloid lineage that opens new opportunities for therapeutic intervention. It is potentially beneficial that overlapping cellular functions also controlled by the CTIP2/p-TEFb repressive complex include many genes that participate in the response to HIV.

Several presentations detailed *in vitro* analyses of chemotherapeutic approaches targeting expression from the latent HIV provirus promoter. Most of these studies compared effects with the pan-HDAC inhibitor (HDACi), vorinostat (SAHA) that targets all four classes of HDAC and has been shown to activate virus transcription in resting CD4+ T-cells in HIV-infected patients on ART in two clinical studies lead by David Margolis [9] and Sharon Lewin (Elliott CROI 2013). Dr. Wightman from the latter group presented an evaluation of newer HDACi’s using a primary T-cell and cell line models of latency for more potent, less toxic and more selective compounds [10]. While all the tested HDACi activated HIV production in latently infected primary T-cells, entinostat and vorinostat had greatest potency and panobinostat, another pan-HDACi, had greatest toxicity. Molecular studies confirmed that entinostat achieved superior viral activation while acting on only HDAC1 among the four HDAC classes in the primary cell model of latency, promising fewer adverse activities. While these studies did not examine effects of these alternative HDACi on latently infected cells from patients, it was reassuring that there were no effects on markers of immune function and identified entinostat, an Ames-test negative HDACi, as a strong future candidate for clinical trial for HIV activation.

Dr. Mahmoudi from Erasmus Medical College, Rotterdam explored how the Wnt pathway promotes an accumulation of β-catenin, which in turn evicts repressors from TCF/LEF-1 sites, as found in the HIV LTR promoter to promote transcription [11]. Treatment of cell line and primary T-cell models of HIV latency with natural ligands of Wnt receptors, Wnt3A and R-spondin, promoted recruitment of TCF/LEF and β-catenin to the LTR and activated latent HIV. Wnt-mediated activation of latent HIV was synergistically augmented with lithium chloride that blocks GSK3β-mediated ubiquitylation and degradation of β-catenin [12], and with the HDACi SAHA.

Mathias Lichterfeld presented data on memory stem cells (T_SCM_), a recently described T cell subset with a naïve-like phenotype, but functional memory characteristics. Although the overall contribution to the CD4 T cell reservoir was low compared with central or effector memory cells, the proportion of T_SCM’s_ infected was higher (although not significantly) and less impacted by ART. Inhibition of the Wnt/ β-catenin pathway - which has been associated with T_SCM_ induction – was used to test impact on the HIV-1 reservoir. The agent PRI-724/C82 (a β-catenin inhibitor) was shown to induce differentiation of T_SCM_ into shorter-lived subsets *ex vivo*, suggesting a possible role in cure strategies. Preliminary data showed synergy between Panobinostat and PRI-724/C82 for both acetylation and intracellular HIV-1 RNA expression in the HIV latently infected ACH-2 cell line. Taken together, the studies on Wnt/ β-catenin pathway highlight opportunities for developing more effective drug cocktails to activate latent HIV and reduce the lifespan of the T_SCM_ reservoir_._ However, potential problems from the strong association of Wnt pathway activation with solid tumour malignancies and other broad cellular activities need to be addressed.

### Intrinsic and adaptive immune control of latent HIV infection

Cellular interactions between T-lymphocytes and HIV infected dendritic cells (DC) are known to promote efficient HIV-1 transmission [13]. Bin Su from the University of Strasbourg, France, showed data that indicates this crosstalk also stimulates HIV-1 replication in DCs by inducing reduced expression of the host restriction factor SAMHD1 that hydrolyses dNTPs to low levels in monocyte derived DCs (mDC). Direct cell-cell contact induces a reduction in DC SAMHD1 to similar low levels as occur following Vpx antagonism during SIV or HIV-2 infection. Therefore, mDC interactions with CD4 T or B cells promote sufficient dNTP levels to support HIV-1 replication. These findings were confirmed and extended by Dr. Xu Yu from the Ragon Institute in Boston, USA, who compared HIV replication and the relative strength of the intrinsic immune responses in mDCs from elite controller patients who have low HIV loads without ART, with chronic progressing and HIV naïve individuals. Stronger cell intrinsic IFN-I responses were observed in mDC from elite controllers (EC) and this was associated with the accumulation of viral RT products and diminished proviral integration. A proposed mechanism was that EC patients expressed significantly lower levels of SAMHD1 and TREX that restrict RT and lower levels of LEDGF/p75 and Transportin 3 that promote integration. It was proposed that accumulating viral intermediates drive elevated type-I IFN production by infected mDCs, and consequently enhanced CD4 and CD8 T-cell responses. Drugs developed to target this DC-lymphocyte crosstalk and the intrinsic sensing of viral products by DCs may interfere with HIV-1 replication in DCs and enhance more highly-effective IFN-stimulated cellular immune responses.

A study from Dr. Ndhlovu from University of Hawaii examined a β-galactoside binding lectin, Galectin-9 (Gal-9), that was proposed to contribute to persistent T-cell activation and HIV entry into resting cells, but also to induce CD4 T cell apoptosis and immunosuppression after binding the Tim-3 receptor [14,15]. Plasma from EC had elevated levels of Gal-9, and CD8+ T-cells had elevated Tim-3 receptor levels compared to ART-suppressed patients, and both were higher than in uninfected patients. Recombinant Gal-9 selectively killed CD8 cells and drove CD4 T-cell responses and this was blocked with α-lactose as a competitive inhibitor, suggesting a testable new therapeutic intervention.

### Characterising the latent HIV reservoir

Sarah Palmer presented longitudinal analyses of single genome sequences exploring the dynamics of the HIV reservoir during suppressive therapy. Patients recruited in both acute and chronic infection and on ART for between 4 to 12 years were sampled from compartments including peripheral blood, bone marrow, gut associated lymphoid tissue and lymph node. Patients were sampled prior to ART and then twice (6 months apart) after suppressive therapy. Dr Palmer reported a substantially higher frequency of infection in patients treated in chronic versus acute infection in all T cell subsets from both blood and GALT. Interestingly, the central/transitional memory cell compartment contained the majority of the reservoir in the peripheral blood (mean 56.3%), whereas in the GALT most infected cells were in the effector memory compartment (mean 68.4%). Phylogenetic analyses based on a Gag-Pol fragment provided no evidence for on-going viral replication during the years of suppressive therapy in any compartment.

Richard Koup presented an analysis of spliced and unspliced HIV RNA in CD4 T cells sorted into bright, dim and null cells. Cells containing single copies of spliced and unspliced RNA transcripts were identified by qPCR, with spliced tat and rev transcripts used to discriminate active viral RNA production from the genomic RNA present in incoming virions. Full-length HIV RNA transcription was associated with CD4 down-regulation, and the greatest numbers of HIV RNA transcripts were found predominantly in activated cells. Labeled monoclonal antibodies against Env were used to sort HIV infected cells. However, although monoclonal antibodies could be used to identify infected T cells in an *in vitro* model (particularly anti-V1V2, VRC07 and PGT121 antibodies), the signal from *ex vivo* index sorted samples was weak making the detection of HIV Env expression using mAbs difficult in these samples.

### Clinical studies exploring the role of ART in HIV cure strategies

#### Treating early in paediatric HIV infection

Deborah Persaud gave a keynote presentation on the impact of ART on the HIV-1 reservoir in the paediatric setting. She defined 'cure’ in children as permanent disease remission off ART, with normal CD4 counts, preservation of immunity to childhood vaccinations and no evidence for immune activation or inflammation. The case of the Mississippi child first reported at the Conference on Retroviruses and Opportunistic Infections (CROI) 2013 was reviewed, with confirmation that the child – now 33 months old – remains in remission with no virological or immunological evidence of HIV-associated pathogenesis [4]. Perinatal infection provides a unique opportunity to determine how early cART might impact reservoirs in neonates when the majority of blood T lymphocytes are a naïve CD45RA phenotype, and possibly less prone to infection [16,17].

Whereas it is clear that HIV-related mortality is high in untreated children (36.8% at one year), early ART started within 3 months saves lives, based on data from the CHER Trial [18], but although early ART restricts the reservoir size, it remains detectable in 60% of cases treated at 2 months [19]. Additionally, early suppressive ART limited viral diversity and resulted in clearance of HIV-1 specific antibodies. In a comparison of children treated early (n = 5) versus late (n = 4), the former had significantly lower reservoirs (using both proviral load quantification (p = 0.048) and IUPM assays (p = 0.008)). After a median of 187 months of ART no replication competent virus could be recovered from the early-treated children in contrast to those treated late (4/4 detectable). The data indicated that treatment in the first few weeks of life made a significant difference compared to those treated after 3 months. However, models of proviral decay suggested that it could take around 20 years for the proviral load to become undetectable in those treated early.

#### The impact of ART in early and chronic adult HIV infection

Laurent Hocqueloux presented data on the timing of ART initiation in chronically infected adults [20,21]. In an analysis of 309 patients with sustained virological suppression followed for a median of 3.7 years, the proportion of patients achieving a composite endpoint of a CD4 count ≥ 900 cells/mm3, a CD4/CD8 ratio > 1 and HIV DNA <2.3 log copy/10^6^ PBMC, after initiating ART in the < 200, 200 – 499, ≥ 500 CD4 count strata were 0% (n = 124), 3% (n = 155) and 30% (n = 30), respectively, reinforcing the importance of the nadir CD4 cell count. In contrast, an analysis of treatment commenced in primary HIV infection (PHI) was presented by Antoine Chéret from the ANRS 147 OPTIPRIM study - a randomized study of tenofovir/emtricitabine and ritonavir-boosted darunavir with or without raltegravir and maraviroc [22,23]. Data from both study arms showed a reduction in HIV DNA of a median -1.34 log copies/10^6^ PBMC between baseline and 12 months in 67 patients. This reduction negatively correlated with baseline plasma HIV RNA and HIV DNA. In the SPARTAC trial a delay in viral rebound had been reported following treatment with a 48-week course of ART in primary HIV infection [24]. James Williams from the University of Oxford presented cross-sectional and longitudinal data from SPARTAC showing a significant decrease in the reservoir after 12 weeks of ART, and with evidence of rapid rebound on stopping therapy. Correlations were shown between both 'total’ and 'integrated’ proviral load measurements at the point of stopping ART (p = 0.017 and p = 0.041, respectively) and the time to the trial primary end-point (of reaching a CD4 cell count of 350 cells/ul or commencing long term ART), confirming the clinical relevance of proviral quantification. Together these studies reinforce the key opportunity that PHI represents for restricting the size of the HIV reservoir with early ART. A key question is whether the rate of proviral decay differs in those treated early versus late, once virus is controlled.

### Clinical studies to activate latent HIV gene expression

Preliminary findings from the CLEAR trial were presented by Dr. Tolstrup from Aarhus University, Denmark. This was a single arm study of panobinostat, a pan-HDAC inhibitor, given as a 20 mg oral dose three times a week, every other week over 8 weeks, to 15 adults on suppressive ART with a CD4 count > 500 cells/mm^3^. Data for the primary endpoint of cell-associated HIV RNA were not available, but a qualitative transcription mediated amplification (TMA) system (PROCLEIX ULTRIO Plus, Genprobe) was presented showing that the proportion of samples which were positive increased from 26% pre-treatment to 60% during the first treatment week (p = 0.009). These may represent the first data of an activating agent leading to plasma release of viral particles, although a more comprehensive analysis is needed. In addition, there was clear evidence of rapid acetylation/deacetylation with each cycle of treatment and good tolerability, with no adverse events greater than grade I, and no significant differences in neutrophil or platelet counts. An optional analytical treatment interruption is planned for all participants.

IL-7 induces HIV production *in vitro* from resting CD4 T cell subsets [25]. The ERAMUNE 01 study was an international, randomized study of 56 weeks of intensification with raltegravir and maraviroc with or without three doses of IL-7 in adults on suppressive ART with CD4 count ≥ 350 cells/mm^3^ and proviral HIV DNA between 10 and 1000 copies/10^6^ PBMC. Twenty-nine participants were randomized and all have completed 80 weeks of follow up. Baseline characteristics were well matched apart from longer duration of ART (median 13 versus 7 years) and higher median baseline HIV DNA (564 versus 287 copies/10^6^ PBMC) in the IL-7 arm. No participant reached the primary endpoint of at least a 0.5 log decrease in proviral HIV DNA in PBMC at 56 weeks. A large increase in CD4+ and CD8+ cells in blood was seen in the IL-7 arm at week 12, which declined thereafter, but remained significantly higher than baseline at week 56. Most of this increase was in central memory T-cells (T_CM_). No significant change in HIV DNA concentration was seen at 56 or 80 weeks in either arm when analysed as copies per 10^6^ PBMC or per 10^6^ CD4 cells, but a significant increase was seen at both time points in the IL-7 arm when analysed as copies per mL of whole blood, due to persistence of the CD4 cell count increase. A trend was seen towards lower HIV DNA in the intensification only arm at week 80 (P = 0.064), but not at earlier time points. The increase in the HIV reservoir in the IL-7 arm would seem to preclude the use of this agent to purge the HIV reservoir.

### Bone marrow transplantation to eliminate the HIV reservoir

Timothy Henrich presented data from two HIV + ve individuals who had received allogeneic stem cell transplants and had subsequently undertaken a treatment interruption. Both recipients were CCR5Δ32 heterozygotes, underwent reduced-intensity conditioning ahead of allogeneic hematopoietic stem cell transplantation from wild type (WT) CCR5 donors and both had significant chronic graft-versus-host disease [26]. Prior to treatment interruption at 4.3 and 2.6 years post-transplant, there was no evidence of HIV infection with negative HIV DNA and viral outgrowth assays. Microchimerism assays demonstrated that donor cells constituted virtually 100% of PBMCs, but the mechanisms driving this were not clear. Both patients were being followed with weekly plasma HIV RNA and biweekly PBMC HIV DNA testing, as well as regular single copy HIV RNA assays and large volume PBMC sampling. At weeks 7 and 14 post-interruption, no HIV plasma RNA or PBMC DNA had been detected, and although longer follow-up is clearly needed, these data were possible early evidence for two further cures. The contrast with the 'Berlin’ patient who received the CCR5∆32 transplant is striking, and raises the intriguing possibility that the graft versus host response rather then the HIV resistance of the transplanted cells that was the key determinant of viral clearance.

### Immunological interventions to target latent HIV infection

Clinical trials testing therapeutic strategies to reactivate latent virus have highlighted the need to concurrently boost immune responses to clear cells expressing antigens from reawakened provirus. The role of dendritic cells (DC) in enhancing this response received attention in three presentations. Kellie Smith from the University of Pittsburgh developed a DC immunotherapy using autologous DC loaded with inactivated HIV-1 derived from the autologous ART reservoir to efficiently prime residual memory T-cells and naïve CD8+ T cells that jointly kill HIV superinfected CD4+ T-cells. These data demonstrated that chronic, untreated HIV-1 infection did not irreparably damage the ability to prime CTL against autologous virus with DC expressing antigens specific for the autologous HIV-1 reservoir, reinforcing the potential for DC-based immunotherapies.

Eric Arts showed that efficient yeast cloning and recombination techniques [27] could be used to prepare replication-incompetent lentivectors expressing autologous viral antigens from plasma taken at the commencement of ART. These vectors were used to present autologous viral antigens on DCs, leading to high-level stimulation of HIV production from lately infected CD4+ T-cells compared to reference strain HIV or other common antigens. This activating vector may also serve as a useful therapeutic vaccine stimulating CTL to clear reactivating HIV, and clinical trials are advancing to test this concept.

An alternative approach developed by Argos Therapeutics uses electroporation to introduce Gag, Nef, Rev and Vpr RNA from autologous HIV into DC, together with RNA expressing CD40L to enhance antigen-specific immune stimulation without CD4+ T-cell help as an immunotherapy concept called AGS-004. Initial clinical studies examining six monthly dosing of AGS-004 gave promising results in reducing viral set point by 1.2 logs and reducing proviral load in some patients.

Una O’Doherty from the University of Pennsylvania presented data using a primary CD4 T cell model of latency in which she has previously reported Gag protein expression [28]. CD4 T cells from elite suppressors were superinfected and targeted with autologous CD8 cells. Killing was evidenced by a drop in Gag + ve 'latent’ cells from 10.3% to 4.4%, which could be blocked with an anti-MHC antibody. In an *in vivo* application of the model, only between 0.1 and 2% of latently infected cells were found to produce Gag, which might be of concern if immunotherapy is to be an integral part of HIV cure.

Dr. Shuzo Matsushita presented data from KD-1002, a phase 1b dose escalation study of KD-247, a neutralizing humanized monoclonal antibody [29,30]. Twenty-seven HIV + ve adults were enrolled off ART, with a CD4 count >350 cells/mm^3^ and a HIV envelope gene sequence encoding the principal neutralizing determinant (V3-tip) corresponding to high KD-247 binding activity. Participants were randomized to one of three dosing cohorts and received active infusion or placebo weekly for three weeks. Mean change from baseline in plasma HIV RNA was in the range of -0.2 and -0.4 log copies/ml for the two larger dose cohorts (8 mg/kg and 16 mg/kg) following the day 8 and day 15 infusions.

J. Victor Garcia took the strategy of targeting the latent reservoir further by using an anti-Env antibody-immunotoxin that delivered Pseudomonas exotoxin to residual HIV infected cells in the humanized mouse model [31]. Administering endotoxin every other day for 14 days to ART treated animals reduced cell-associated HIV RNA production over ART alone. This approach showed the susceptibility of the residual active HIV reservoir to targeted cytotoxic therapy, and HIV–targeted immunotoxins might compliment combination chemotherapy.

### Ethics, regulatory issues and product development

Two roundtable panel sessions discussed key issues from the diverse perspectives of the different stakeholders. The first panel assembled HIV cure clinical trialists, pharmaceutical industry representatives and community ethicists^a^ to discuss issues that will accelerate product development and regulatory agency approval for a rapid and safe transition into the clinic of HIV cure therapies having strong end-user acceptance and wide accessibility for all patient populations. Open discussion with relevant regulatory authorities was advised to clarify the endpoints measured and the risk-benefit criteria required for an accelerated approval. Appropriate endpoints are especially important for combination cure therapies, such as a small molecule inhibitor together with a biologic and a vaccine. Although analytical treatment interruption (ATI) was previously considered unacceptable, it is now a valid option for cure strategies in appropriate well-controlled patient populations to minimise the potential risk of viral rebound. At these early stages of clinical cure investigation, it is unlikely that research of blood samples will be sufficient, and tissue sampling will also be necessary, at least for a portion of study participants, to gain insight into biomarkers for long-term control.

The second roundtable discussion on ethics and patient expectations provided a timely reminder of the need to address the biomedical question of HIV cure in parallel to the social and ethical questions. The panellists^b^, who included scientists and clinicians involved in cure research, community members, research funders and bioethics specialists, resolved that the scientific excitement for a potential HIV cure should not detract attention from the financial, social, ethical and moral implications of cure research. Many of the ethical considerations for HIV cure research have been recently reviewed [32] and it is clear that no standard set of ethical rules can apply to the entire field of cure research. As for many HIV research areas, research towards cure should be carried out in concert with HIV patients. A deeper and meaningful understanding of patient expectations and willingness to participate in HIV cure studies will be paramount to the success of the field.

## Conclusion

The “Towards a Cure” symposium captured the increasing research momentum based on the growing scientific understanding of the HIV reservoir and the increasing evidence from cases of HIV remission or cure. There is increasing interest in chemotherapeutic interventions promoting viral remission, and the first small steps have been encouraging. Early data from clinical studies with HDACi showed they were safe, well tolerated and successfully stimulated unspliced HIV RNA as the primary endpoint, providing optimism for broadly applicable viral reactivation strategies. However, viral RNA has not yet been linked with viral protein expression, and reduction in proviral load. Other biomarkers are needed that better correlate with elimination of the viral reservoir during curative interventions. Importantly, more HIV-specific activating drugs are following and these offer better specificity for HIV induction, and may potentially team with therapeutic vaccines or other strategies to eliminate cells containing reawakened HIV. But there is clearly a long way to go and key challenges lie in the search for combination interventions that not only lead to activation of latent HIV infection, but also significant decreases in the size of the HIV reservoir.

Evidence is mounting that early implementation of ART can assist in achieving viral control off therapy, possibly by preventing the seeding of virus into reservoirs that endure for the life of the patient. It remains to be resolved if the reservoir seeded during chronic infection differs from that laid down in the primary infection, and if it can be eliminated by chemotherapeutic intervention.

There are still large gaps in our understanding of the molecular mechanisms that restrict the expression and release of replication competent HIV from the different tissue reservoirs. While restrictive epigenetic and transcriptional programs largely underpin the limited HIV expression from most residual integrated proviruses, it is clear that various other molecular mechanisms can reversibly restrict viral expression, producing a diverse reservoir of residual latent provirus. An increasingly important impediment in the field is access to validated methods to measure the true size of the relevant replication competent latent virus reservoir.

The terms “sterilising” and “functional” cure have been used to describe patients who have achieved complete eradication or who control viral replication in the absence of ART, respectively [33]. Whereas the two concepts are valid, the meaning of 'functional’ is unclear and - recognising the clear parallels with cancer therapeutics – the term “HIV remission without ART” has been proposed. The precise definition 'remission’ still needs to be agreed, for example the duration of undetectable viraemia required or the risk of transmission.

HIV cure research has benefited from strong interactions between basic scientists and clinicians, but it is clear that important insights provided by social scientists and members of the HIV community are required to determine the expectations surrounding HIV cure and the acceptability of different curative strategies. A trans-disciplinary approach must include non-HIV experts to bring new insights from the fields of oncology, other chronic infections and inflammatory diseases. Wide consultation is also needed to identify the appropriate conditions for interruption of ART and the protocols for monitoring viral recrudescence during treatment interruptions and the thresholds for reinstating therapy.

## Endnotes

^a^Roundtable panelists: David Margolis, University of North Carolina at Chapel Hill (Chair); Guenter Kraus, Tibotec/Janssen; Veronica Miller, The Forum for HIV collaborative research; Bill Whittaker, National Association of People living with HIV Australia; Hiroyu Hatano, University of California San Francisco.

^b^Jintanat Ananworanich, The Thai Red Cross AIDS Research Centre (chair), Jeremy Sugarman (Johns Hopkins Berman Institute of Bioethics, United States), Laurindo Garcia (B-Change), Eric Fleutelot (Sidaction), Bruno Spire (Inserm), Rowena Johnston (amfAR), Sharon Lewin (Alfred Hospital, Monash University, Burnet Institute).

## Competing interests

The authors declare that they have no competing interests.

## Authors’ contributions

All authors contributed to writing these critiques, and all authors read and approved the final manuscript.
